# Operant conditioning in antlion larvae and its impairment following exposure to elevated temperatures

**DOI:** 10.1007/s10071-021-01570-9

**Published:** 2021-10-24

**Authors:** Krzysztof Miler, Inon Scharf

**Affiliations:** 1 Institute of Systematics and Evolution of Animals, Polish Academy of Sciences, Kraków, Poland; 2 School of Zoology, Faculty of Life Sciences, Tel Aviv University, Tel Aviv, Israel

**Keywords:** Climate change, Cognition, Heat, Memory, *Myrmeleon hyalinus*, Spatial learning, T-maze, Thermal stress

## Abstract

Although ambush predators were previously considered limited in their cognitive abilities compared to their widely foraging relatives, there is accumulating evidence it does not hold true. Pit-building antlions are already known to associate vibrations in the sand with the arrival of prey. We used a T-maze and successfully trained antlions to turn right or left against their initial turning bias, leading to a suitable substrate for digging traps. We present here the first evidence for operant conditioning and T-maze solving in antlions. Furthermore, we show that exposure of second instar larvae to an elevated temperature led to impaired retention of what was learned in a T-maze when tested after moulting into the third instar, compared to larvae raised under a more benign temperature. We suggest that climate change, involving an increase in mean temperatures as well as rare events (e.g., heatwaves) might negatively affect the retention of operant conditioning in antlions, alongside known, more frequently studied effects, such as changes in body size and distribution.

**Supplementary Information:**

The online version contains supplementary material available at 10.1007/s10071-021-01570-9.

## Introduction

Operant learning is a reversible process in which “reinforcers”, either rewards or punishments, shape behaviour (Staddon and Cerutti 2003). In short, it is a process in which actions increase in probability after positive consequences and decrease in probability after negative ones. Learning is affected by the conditions under which animals learn and their physiological state. Stress, or the animal responses to exposure to a harsh environment or situation (Stott 1981), strongly modifies behaviour and learning performance in particular. Whereas it is accepted that major stress should have a negative impact on behaviour, moderate stress may contribute to performance, in a process termed hormesis (Le Bourg 2007; Cutler 2013). Similarly, activity and learning performance reach their peak following moderate starvation and intrinsic stress (‘stress hormones’), respectively, resulting in an inverted U-shaped link between stress level and learning performance or activity (Sandi and Pinelo-Nava 2007; Scharf 2016).

Considering the ongoing anthropogenic changes, stressors may act on animals increasingly often and lead to modifications in their behaviour (Beever et al. 2017; Baxter-Gilbert et al. 2019; Buchholz et al. 2019). High temperatures and heatwaves have become more common in recent years (Russo et al. 2014). July 2021 was the hottest month on record for the planet (National Oceanic and Atmospheric Administration 2021), which illustrates the timeliness of the topic. The mechanisms by which thermal stress affects neural development and cognitive processes, such as learning in animals, are still not well understood (Buchanan et al. 2013). Its effect is nevertheless important regardless of the mechanism and there is still little known about how thermal stress affects animal cognition (Soravia et al. 2021).

The learning ability of ambush predators, which sit-and-wait at a suitable site and hunt prey that enters their attack range, has been suggested to be limited compared to more active related species (Huey and Pianka 1981; Day et al. 1999). That said, it is clear that ambush predators make decisions that require behavioural flexibility, sensitivity to exterior conditions and their own physiological state, and thus need to monitor changes in their immediate environments. Trap-building predators are a sub-group of ambush predators, which construct traps to hunt prey. They either construct the trap with self-produced materials, such as web-building spiders or create it using substrates available in their environment, such as pit-building antlions or wormlions. Antlion larvae (Neuroptera: Myrmeleontidae) dig pitfall traps in loose soil and ambush arthropods. They spend the vast majority of their long life-cycle as larvae and the adults are short-lived (Scharf and Ovadia 2006; Scharf et al. 2010).

Antlions demonstrate active microhabitat selection according to multiple factors, such as preferred temperature, shade level, soil particle size, or soil dryness (Scharf et al. 2008a; Barkae et al. 2014; Devetak and Arnett 2015; Miler et al. 2019). Their behaviour is flexible, for example in relocating their traps when prey arrivals stop abruptly but remaining longer in the same site when prey availability decreases gradually, or in changing pit dimensions to catch prey of different sizes (Jenkins 1994; Lomáscolo and Farji-Brener 2001). They are capable of associating vibrations, to which they are highly sensitive, with prey arrival, which results in increased hunting success and growth (Guillette et al. 2009; Hollis et al. 2011; Kuszewska et al. 2016).

Although antlions, through associative processes, can learn to react to vibrations when linked with prey arrival, it is unknown whether they are capable of other learning types, such as operant conditioning. Furthermore, antlions are frequently exposed to heatwaves as they are ambush predators of low mobility, common in warm environments. High temperature affects various aspects of behaviour and life-history of antlions (Scharf et al. 2008b, 2009; Klokočovnik et al. 2016; Miler et al. 2020), but no studies have examined the effect of heat on any type of learning in these insects. Here, we address operant conditioning and the effect of heat on its retention by studying how the thermal conditions experienced by the larvae during one developmental stage affect learning retention at a later one. Because we were interested in operant conditioning rather than other types of learning, we first established a new methodology inspired by classical studies in *Tenebrio molitor* larvae (Alloway and Routtenberg 1967; Alloway 1972). The method comprises repeated trials in a T-maze, where antlions choosing one of the sides are rewarded with sand, allowing them to dig in, whereas those reaching the opposite side fall on a table and are then returned to the maze start point. T-mazes are common in behavioural research and have been used to study different learning types (Skinner et al. 2003; Jakob et al. 2007; Czaczkes 2018), but have been never applied in any species of pit-building predators. Based on their previously demonstrated behavioural flexibility and learning ability, we predicted that antlions would be capable of operant conditioning. We also expected antlions reared under an elevated temperature to retain memory shorter than those reared under a benign temperature. The reason may be either the stress induced by a higher temperature or the faster metabolism, leading to faster behavioural change.

## Methods

### Animal capture and husbandry

In June 2021, we collected second-instar larvae of *Myrmeleon hyalinus* antlions (Israel, Tel Aviv, coordinates: 32°06′42′′ N, 34°46′46′′ E), identified following Badano and Pantaleoni (2014). We then housed them individually in the laboratory (air-conditioned room, constant 25 °C, natural non-direct illumination) in plastic cups (5.5 cm in diameter, 7.2 cm in height) half-filled with sand (to the height of ~ 4 cm; sieved construction sand with particle size ranging 250–710 µm). A temperature of 25 °C has proven to be a suitable temperature to keep the antlions at and conduct behavioural experiments (Scharf et al. 2008a; Alcalay et al. 2015). We left the animals for a week to acclimate, during which we fed them once with a single *Tribolium castaneum* larva (here and throughout, mean ± SD: 27.8 ± 3.6 mg, *N* = 10) to standardize hunger level.

### Experimental setup

We used the T-maze method to study operant conditioning and applied it to antlion larvae, which has not been done before. We performed two laboratory experiments. In the first experiment, we established a new method to examine operant conditioning in antlions. In the second experiment, we examined whether development at benign or elevated temperatures produced differences in learning ability in antlions. We used 20 individuals in the first experiment and 80 individuals in the second experiment. To prevent any bias in the results due to differences in body mass, we allocated larvae to different groups according to body mass after weighing them to the nearest 0.1 mg using an electronic scale (first experiment: mean ± SD: 63.4 ± 13.4 mg; second experiment: 36.4 ± 17.0 mg). In the first experiment, we created 10 sets of two larvae with similar body masses. We allocated one larva from each set to one of the two treatments. The two treatments did not differ in the body mass of larvae as tested by a Welch two-sample *t* test (*t =* 0.130, *p =* 0.898). In the second experiment, we created 40 sets of two larvae with similar body masses. We allocated one larva from each set to one of the two maintenance temperatures. The two maintenance temperatures did not differ in the body mass of larvae as tested by a Welch two-sample *t* test (*t =* 0.279, *p =* 0.781). In both experiments, we housed all larvae in the plastic cups used during the acclimation period and labelled those with the individual ID.

### The first experiment: examination of operant conditioning

**Fig. 1:**
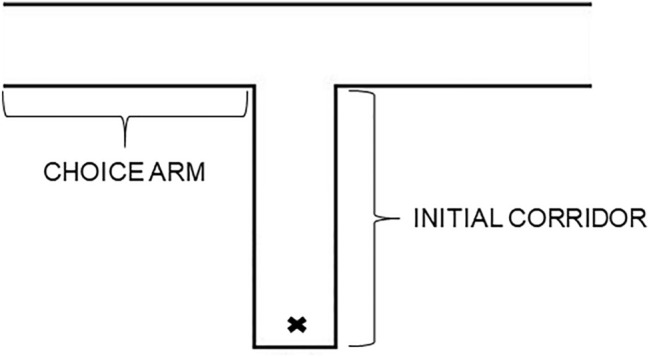
Scheme of the T-maze used in the study. Symbol ‘x’ marks the starting point. The scheme is not to scale. Length of the initial corridor: 10 cm, length of each choice arm: 34 cm, width: 1.6 cm, height: 2 cm. One arm of the maze led to sand, enabling the antlions to dig in, and the other led to a void

After weighing and allocating the larvae to the learning treatment or the control, we performed an initial turning preference test for all larvae. On that day, we tested each larva five times, with approximately an hour inter-trial interval. For each trial, we used a T-maze made of polypropylene plastic sheet, cut to size and with its walls and floors glued together using hot glue (length of the initial corridor: 10 cm, length of each choice arm: 34 cm, width: 1.6 cm, height: 2 cm; Fig. 1). Each trial commenced by releasing the larva at the beginning of the initial corridor and lasted until it reached the end of one of the choice arms. Then, we placed the plastic cup of the larva under that choice arm, for it to drop into. We positioned the maze 10 cm above the table, on a plastic box, to easily perform this manipulation. The distance from the end of the choice arm to the surface of the sand in the plastic cup was about 6 cm. We noted whether the larva chose the left or the right choice arm during each trial. After completing five trials, we determined each individual’s initial turning bias by choosing the side each larva turned to three or more out of five trials. The two treatments did not differ in the initial turning preference as tested by Fisher's exact test (*p =* 0.370, 7 individuals turning right in the control treatment and 4 individuals turning right in the learning treatment). We then left the larvae inside their cups for one night.

We performed the training on the next 4 days. We performed five training trials during each training day, with an hour interval between successive trials (20 trials per individual in total). We fed all larvae a single *T. castaneum* larva and left them feeding for ~ 30 min before beginning the first training trial each day. We used our T-maze in each trial and released the larva at the beginning of the initial corridor as in the initial turning preference test.

We tested larvae in two treatments. In the control treatment, we placed the cup of the larva under the choice arm it turned to, for it to drop into. In other words, during the trial, whatever choice each larva in the control treatment made, it always ended up in its cup. We scored each choice using a 0/1 scale (“0”—in line with initial turning preference, “1”—against initial turning preference). In the learning treatment, we placed the cup of the larva under the choice arm that was against its initial turning preference. For example, for a larva with an initial turning preference to the right, during each training trial, we placed its cup under the left choice arm, while under the right choice arm, there was void. In other words, for each larva in the learning treatment, to end up in its cup, it had to choose against its initial preference. If it chose the void, then it dropped 10 cm to the table, from where we retrieved it after a few seconds using entomological forceps and placed it immediately at the beginning of the initial corridor for another, forced trial. We scored each choice using our 0/1 scale (“0”—in line with initial turning preference, “1”—against initial turning preference). In the forced (and unscored) trial, we blocked the choice arm that was in line with the initial turning preference of the larva using a fitted piece of polypropylene plastic sheet. Thus, in the forced trial, the larva had to turn against its initial turning preference to finally end up in its cup and for the trial to stop. Larvae probably perceived the table surface as stressful and ending up in the cup enabled them to bury themselves in the sand, which they always did without delay after reaching the cup. The reward, therefore, is being able to dig in the sand and the situation of not being able to do so is stressful. In total, we performed 20 scored training trials for each larva, with additional unscored, forced trials in the learning treatment. We performed all initial preference trials and training trials in the laboratory (25 °C).

#### Statistical analysis

We analysed the data using R version 4.1.1 (R Core Team 2021). We used a generalized linear mixed model with a binomial distribution to compare the number of choices against initial turning preference during training (lme4 package). We included in the model a fixed factor of treatment (control vs. learning) and a covariate of trial number (1–20) and their interaction. We also included the ID of the larvae as a random factor.

### The second experiment: the effect of temperature on operant conditioning

After weighing the larvae, we placed them in thermal cabinets, set to one of two constant temperatures (25 °C or 29 °C for the benign or elevated maintenance temperatures, respectively) and natural light cycle. We fed these larvae each day with a single *T. castaneum* larva if they were active, similar to Miler et al. (2020). We considered active larvae to be those that (1) maintained functional pitfall traps and (2) were visible with their mandibles protruding from the bottom of the trap. We checked each inactive larva for signs of life or moulting by digging it out. We left live inactive larvae in their cups and discarded dead larvae (8 and 6 individuals died in the benign and elevated maintenance temperatures, respectively, see below on for an analysis). We weighed the larvae that reached the third instar stage using an electronic balance (accuracy of 0.1 mg) to obtain the body mass of moulted larvae. We alternately assigned moulted larvae to one of the two treatments, the learning treatment or the control.

We performed the same procedure on these larvae as detailed for the first experiment: we assessed the initial turning preference of each individual and then performed the training consisting of 20 scored trials. Under the benign and elevated maintenance temperatures, 32 and 34 individuals reached this stage of the experiment, respectively. The two treatments did not differ in the initial turning preference as tested by Fisher’s exact test in the benign temperature (*p =* 0.315, 12 individuals turning right in the control treatment and 9 individuals turning right in the learning treatment) and in the elevated temperature (*p =* 1.000, 12 individuals turning right in the control treatment and 12 individuals turning right in the learning treatment). In addition, 14 days after the last training trial for each individual, we performed a retention test, in which each larva was tested once in the T-maze and scored on our 0/1 scale (“0”—in line with initial turning preference, “1”—against initial turning preference). We performed all initial turning preference trials, all training trials, and the retention test in the laboratory (25°C). The larvae were left unfed and undisturbed in the laboratory during 2 weeks in-between the last training trial and the retention test.

#### Statistical analysis

We analysed the data using R version 4.1.1 (R Core Team 2021). We compared the mortality of antlions using a generalized linear model with a binomial distribution, a fixed maintenance temperature factor (benign vs. elevated), an initial body mass covariate, and their interaction (stats package). We used general linear models including data only from individuals who remained alive to assess the number of days until moulting occurred and the body mass of moulted larvae (stats package). In both models, we included a fixed maintenance temperature factor (benign vs. elevated), an initial body mass covariate, and their interaction. We used a generalized linear mixed model with a binomial distribution to compare the number of choices against initial turning preference during training (lme4 package). In the model, we included two fixed factors, treatment (control vs. learning) and maintenance temperature (benign vs. elevated), a trial number covariate (1–20), and their interactions. We also included a random factor, the ID of the larvae. Finally, we used a general linear model to assess the number of choices against initial turning preference during the retention test (stats package). We included two fixed factors in the model: treatment (control vs. learning) and maintenance temperature (benign vs. elevated) as well as their interaction.

## Results

### The first experiment: examination of operant conditioning

**Fig. 2:**
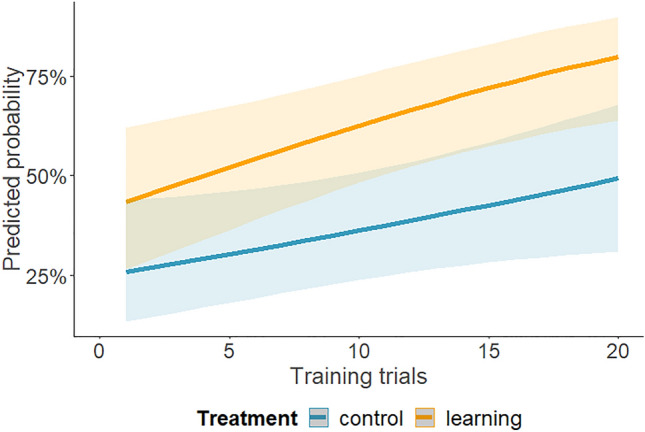
Probability of turning against the initial preference in the control and learning treatment in relation to the number of training trials. Solid lines show the predicted values calculated by the model, with shading indicating estimated 95% confidence intervals

The analysis revealed that both the treatment (*χ*^2^ = 6.43, *p =* 0.040) and the trial number (*χ*^2^ = 13.97, *p <* 0.001) had a significant effect on the number of choices against initial turning preference during training. In both treatments, the effect of the trial number was in the same direction (non-significant interaction, *χ*^2^ = 0.67, *p =* 0.414). As larvae progressed through the training trials, they turned against the initial turning preference more often in both treatments, but it occurred more frequently in the learning treatment (an increase in the probability of up to ~ 50% in the control treatment and ~80% in the learning treatment, Fig. 2). This indicated that operant conditioning took place and the method was successful in discovering it. It further indicated that including the control treatment was important, due to spontaneous increase in turning against initial preference with progressing training trials, even in the absence of training.

### The second experiment: the effect of temperature on operant conditioning

**Fig. 3:**
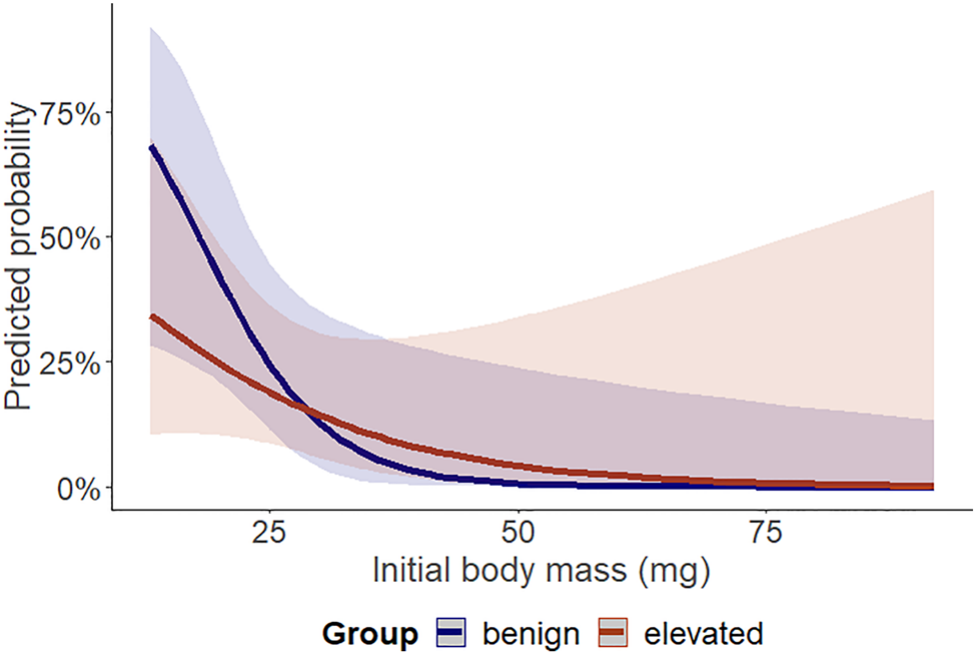
Probability of death in the benign and elevated maintenance temperature (25°C and 29 °C, respectively) in relation to the initial body mass. Solid lines show the predicted values calculated by the model, with shading indicating estimated 95% confidence intervals

The analysis of mortality revealed that the maintenance temperature was not significant (*χ*^2^ = 0.35, *p =* 0.556), but larger larvae were less likely to die than smaller ones (*χ*^2^ = 11.69, *p <* 0.001). Furthermore, the interaction between the maintenance temperature and initial body mass was not significant (*χ*^2^ = 1.15, *p =* 0.286), indicating that the effect of the initial body mass of larvae on mortality was similar under both maintenance temperatures (Fig. 3).

**Fig. 4:**
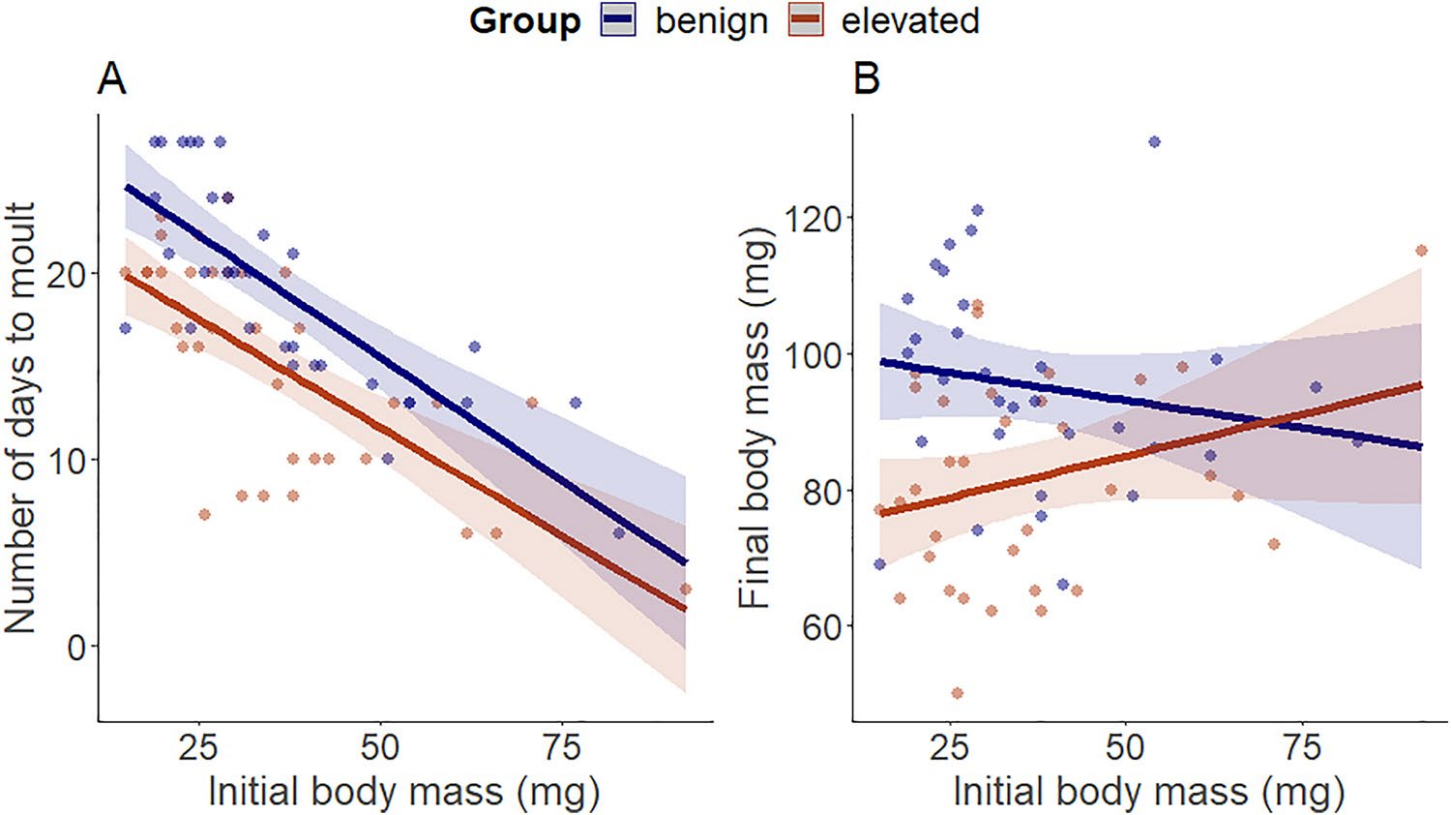
**A** Number of days to moult in the benign and elevated maintenance temperature (25 °C and 29 °C, respectively) in relation to the initial body mass. **B** Body mass after development under the benign and elevated maintenance temperature in relation to the initial body mass. Solid lines show the predicted values calculated by the model, with shading indicating estimated 95% confidence intervals

Among the individuals who remained alive, moulting occurred earlier under the elevated than benign temperature (*F*_1,64_ = 16.148, *p <* 0.001). Larger larvae moulted sooner than smaller larvae (*F*_1,63_ = 74.808, *p <* 0.001, Fig. 4A) under both temperatures, as the interaction between the maintenance temperature and initial body mass was not significant (*F*_1,62_ = 0.283, *p =* 0.596). Regarding the body mass of moulted larvae, individuals at the benign temperature reached larger body masses than those under the elevated temperature (*F*_1,64_ = 13.489, *p <* 0.001). There was no effect of the initial body mass on the mass after moulting (*F*_1,63_ = 0.238, *p =* 0.627) and the interaction between the temperature and initial body mass was not significant (*F*_1,62_ = 3.378, *p =* 0.071, Fig. 4B). Thus, all larvae regardless of their size reacted similarly to different temperatures.

**Fig. 5:**
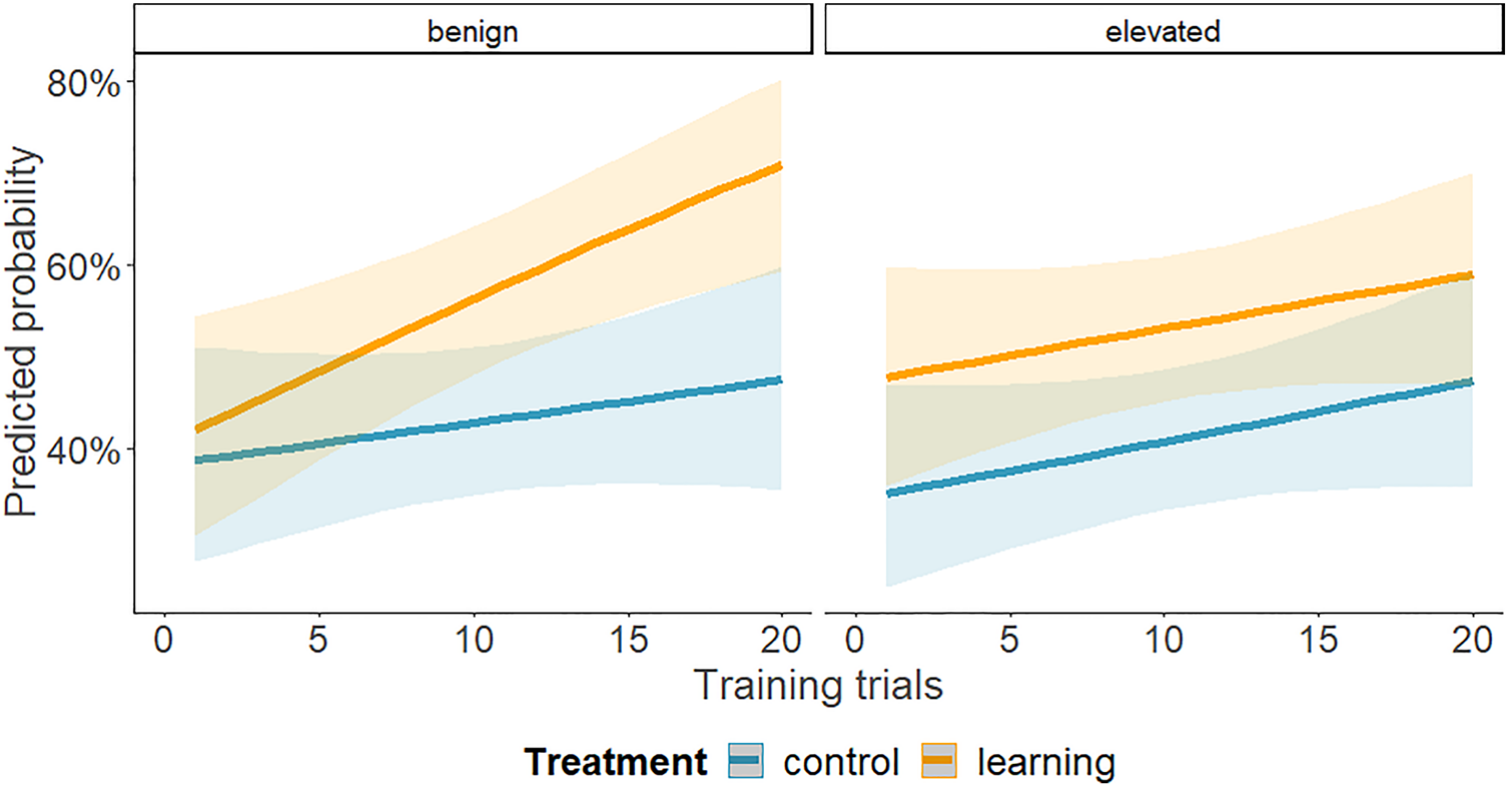
Probability of turning against initial preference in the control and learning treatment in relation to the number of training trials, in larvae originating from the benign or elevated maintenance temperatures (left or right panel, respectively; 25 °C and 29 °C, respectively). Solid lines show the predicted values calculated by the model, with shading indicating estimated 95% confidence intervals

**Fig. 6:**
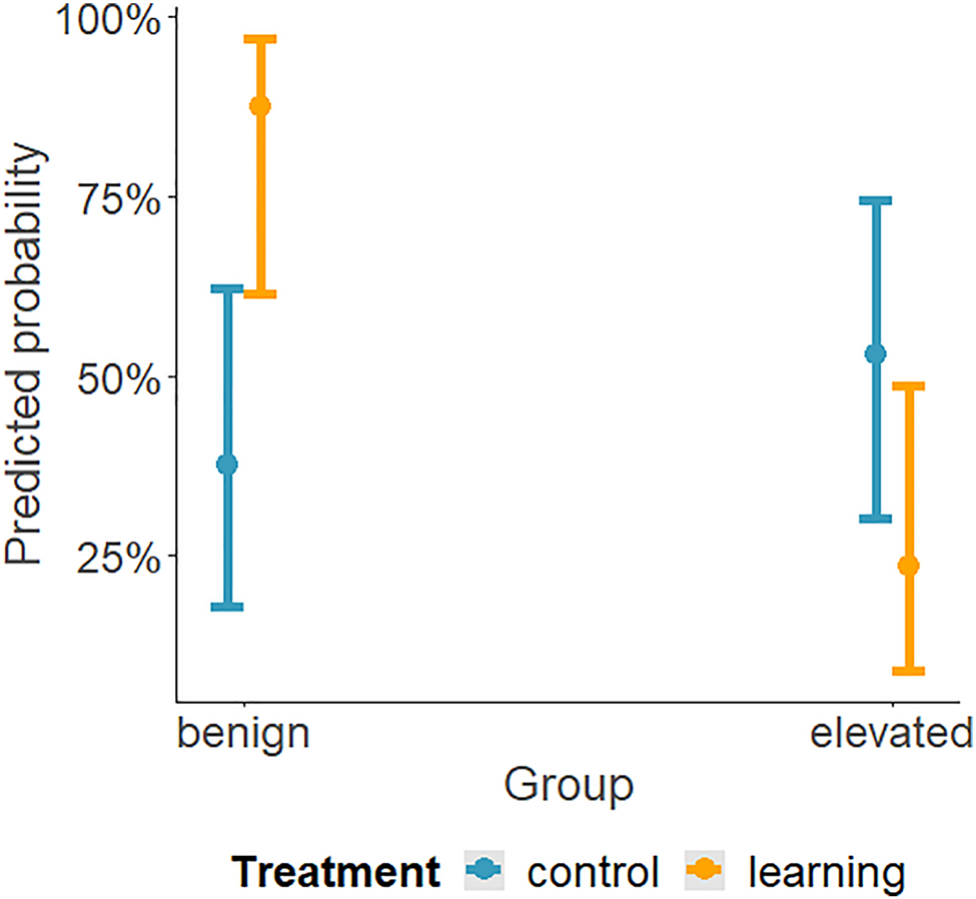
Probability of turning against initial preference during the retention test in the control and learning treatment in larvae originating from the benign or elevated maintenance temperatures. Dots represent the mean probability predicted by the model, with error bars indicating estimated 95% confidence intervals

The analysis of the number of choices against initial turning preference during training revealed that the trial number (*χ*^2^ = 14.11, *p =* 0.015) and the learning treatment (*χ*^2^ = 12.63, *p =* 0.027) were significant. The maintenance temperature (*χ*^2^ = 3.54, *p =* 0.617), as well as all interactions, were not significant (*p >* 0.232 for all interactions tested). Individuals originating from both temperatures turned against their initial preference more often as they progressed through the training trials, and it occurred more frequently in the learning treatment (Fig. 5), resembling the results of the first experiment. Indeed, in the benign temperature, there was an increase in the probability of up to ~ 45% in the control treatment and ~ 70% in the learning treatment, while in the elevated temperature, there was an increase of up to ~ 45% in the control treatment and ~60% in the learning treatment. Even though it seemed that in the benign temperature this increase in turning against the initial preference was more rapid (Fig. 5), this conclusion was not supported by the results of the model (as evidenced by the non-significant interaction, mentioned above). However, the final analysis of the number of choices against the initial turning preference during the retention test revealed that there was a significant interaction between the maintenance temperature and treatment (*χ*^2^ = 11.71, *p <* 0.001, Fig. 6). Specifically, only individuals in the learning treatment from the benign temperature had a high probability of turning against the initial preference 2 weeks after the training, which provides evidence of better retention. This probability of the learning treatment at the benign temperature reached ~ 85%. In contrast, individuals in the control treatment originating from the benign temperature demonstrated a probability of ~ 35%. In individuals originating from the elevated temperature, the probability was about 50% in the control and 25% in the learning treatment. There was no effect of the treatment alone (*χ*^2^ = 0.55, *p =* 0.460) and the maintenance temperature alone was on the verge of significance (*χ*^2^ = 3.96, *p =* 0.047).

## Discussion

We present here a novel and simple method to study operant conditioning in antlion larvae and examine it and its retention in larvae that developed at different temperatures. Although it is already known that antlions can learn to associate vibrations, caused by dropping sand, with the immediate arrival of prey, this is the first demonstration of operant conditioning in these trap-building predators. Exposing the second instar larvae to the elevated temperature seemed not to impair learning compared to the benign temperature, but it did impair retention (cf. Figs. 5 and 6).

The first experiment supported our prediction that antlions should be capable of operant conditioning. The reward we used, the ability to bury itself in the sand, is probably a strong reinforcer in antlions. Antlions keep moving on hard surfaces until they reach loose soil, in which they bury themselves and stop moving. Being buried in sufficiently deep sand probably provides antlions with protection against their own predators (Loria et al. 2008). Future research should evaluate the benefits of operant conditioning, in terms of any fitness proxy, such as hunting success, growth, and predation avoidance. Although many studies have pointed to the ability of insects to learn, the number of studies providing evidence for its fitness benefits is low (but see, e.g., Dukas and Bernays 2000; Hollis et al. 2011).

We believe that our method of operant conditioning may be more accurate than former tests of associative learning in antlions (Guillette et al. 2009; Hollis et al. 2011; Miler et al. 2017). In the latter method, artificially evoked substrate vibrations, caused by sand particles falling next to the antlion pitfall trap, were linked with the arrival of prey, which was dropped into the trap. The weakness of this method is the need for fine standardization and very careful observation of the antlion, such as its mandible movement in response to the vibration cues (Guillette et al. 2009; Miler et al. 2018). These responses might occur spontaneously, without initial training (Devetak 2014), and are sometimes ambiguous. The response variable in our method, in contrast, i.e., turning right or left and falling into a cup, is much more easily observed, and is more comparable to the response variables measured in other arthropods. Future studies should examine whether antlions learned exactly where the sand is (“place learners”) or learned the turning response leading to the desired outcome (“response learners”). Rats, for example, do both (they first learn places and then switch to responses; Packard and McGaugh 1996).

Developing through the second instar under an elevated temperature impaired memory of what was learned after moulting into the third instar, suggesting that the elevated temperature was too stressful for the antlions. Other types of learning impairment occurred in fruit flies and velvet geckos. Animals that experienced elevated temperature during development, as larvae or embryos (respectively), performed poorly on a learning test as adults (Wang et al. 2007; Dayananda and Webb 2017; Abayarathna and Webb 2020). The detrimental effect of elevated temperature on cognitive ability is, however, equivocal and other studies demonstrated the opposite. For instance, exposing three-lined skinks, bearded dragons, and Port Jackson sharks to elevated temperatures at the egg stage enhanced the future cognitive performance of adults (Amiel and Shine 2012; Amiel et al. 2014; Clark et al. 2016; Siviter et al. 2017; Vila Pouca et al. 2019). The reasons for these discrepancies are not clear. On top of examining different cognitive abilities, there could be an inverted U-shaped link between stress level and performance or some hormetic effect of stress (Sandi and Pinelo-Nava 2007; Cutler and Guedes 2017). In addition, the “elevated temperature” used in some experiments could be too low to be indeed stressful (sensu Abayarathna and Webb 2020).

Although we have no accurate data on the thermal preference of the antlion species studied here, our elevated temperature of 29 °C is likely high and stressful for antlions, collected from a Mediterranean (and not a desert) population, which prefers shaded over sunny microhabitats (Scharf et al. 2008c). Previous studies on the same species demonstrated the strong effect of elevated temperature on development, body size, and behaviour (Scharf et al. 2008b; Alcalay et al. 2014). Similar to the current results, antlions raised under higher temperature grew faster but reached smaller size (Scharf et al. 2008b), fitting the ‘temperature-size rule’ of ectotherms (Atkinson 1994; Kozłowski et al. 2004).

Future studies should compare the cognitive abilities of the Mediterranean antlion populations with desert ones. It is possible that in addition to the known behavioural, morphological, and life-history differences among populations of the studied antlion, these populations differ as well in cognitive ability and the effect of temperature on cognition. Differences in learning ability among populations are understudied, although, migratory white-crowned sparrows learnt better than sedentary sparrows (Nelson et al. 1996). Another good question is whether poorer retention in the tested antlions originating from elevated temperatures is adaptive or not (Kraemer and Golding 1997). It could be adaptive if, for example, the habitat changes faster at high temperatures and stored information becomes irrelevant sooner in such conditions. Alternatively, high temperature accelerates metabolism and forgetting might be simply a by-product of the latter.

Antlions tended to turn against their initial turning preference with successive trials, even without any difference between the two arms of the T-maze (Figs. 2 and 5). Changing an initial turning bias could reflect an attempt to escape the maze, as the trial setup likely caused some stress leading to an escape response. By the end of the first and second experiments, individuals from the control treatment were equally likely to turn into each arm. Susceptibility of this bias to repeated testing, as we show here, suggests that turning behaviour may be inconsistent over time (see also Roche et al. 2020). Alternatively, the initial preference might have been a matter of sampling error or random deviations from an even probability. Over trials in the control, there is a regression back to the norm of a 50% chance to turn in any direction.

Climate change comprises both an increase in mean temperatures and its variance, as well as the frequency of rare events. Here, we show that heat might result in memory impairment. This finding expands our current knowledge about the multifaceted effects of heat on antlion larvae (Alcalay et al. 2014; Miler et al. 2020; Miler and Czarnoleski 2021). Antlions are capable of behavioural thermoregulation (Green 1955; Marsh 1987; van Zyl et al. 1996; Abraham 2003) and similar to other taxa may likely adjust to heat by changing their microhabitat utilization, activity time, or hunting strategy (Ma et al. 2018; Johnson et al. 2020; Gotcha et al. 2021). This is nevertheless uncertain and heat stress might still have negative consequences. Considering the fitness benefits of learning under natural conditions (Guillette et al. 2009; Hollis et al. 2011; Kuszewska et al. 2016), its impairment due to heat exposure might translate into a higher risk of failing to reach adulthood or at least in slower development, smaller adult size, or diminished reproduction. The interactive effects of heat and starvation stress on learning are also of interest, as heat elevates metabolism and may lead to faster starvation in the absence of prey.

## Supplementary Information

Below is the link to the electronic supplementary material.

Supplementary file1 (XLSX 54 kb)

## Data Availability

All data generated or analysed during this study are included in this published article and its supplementary information files.
